# Supplementary material for: Setd2 inactivation sensitizes lung adenocarcinoma to inhibitors of oxidative respiration and mTORC1 signaling
Source: Commun Biol. 2023 Mar 10;6:255. doi: 10.1038/s42003-023-04618-3 (PMC10006211; doi:10.1038/s42003-023-04618-3)
Supplement: Supplementary file 2 — Supplementary Information [file 42003_2023_4618_MOESM2_ESM.pdf]

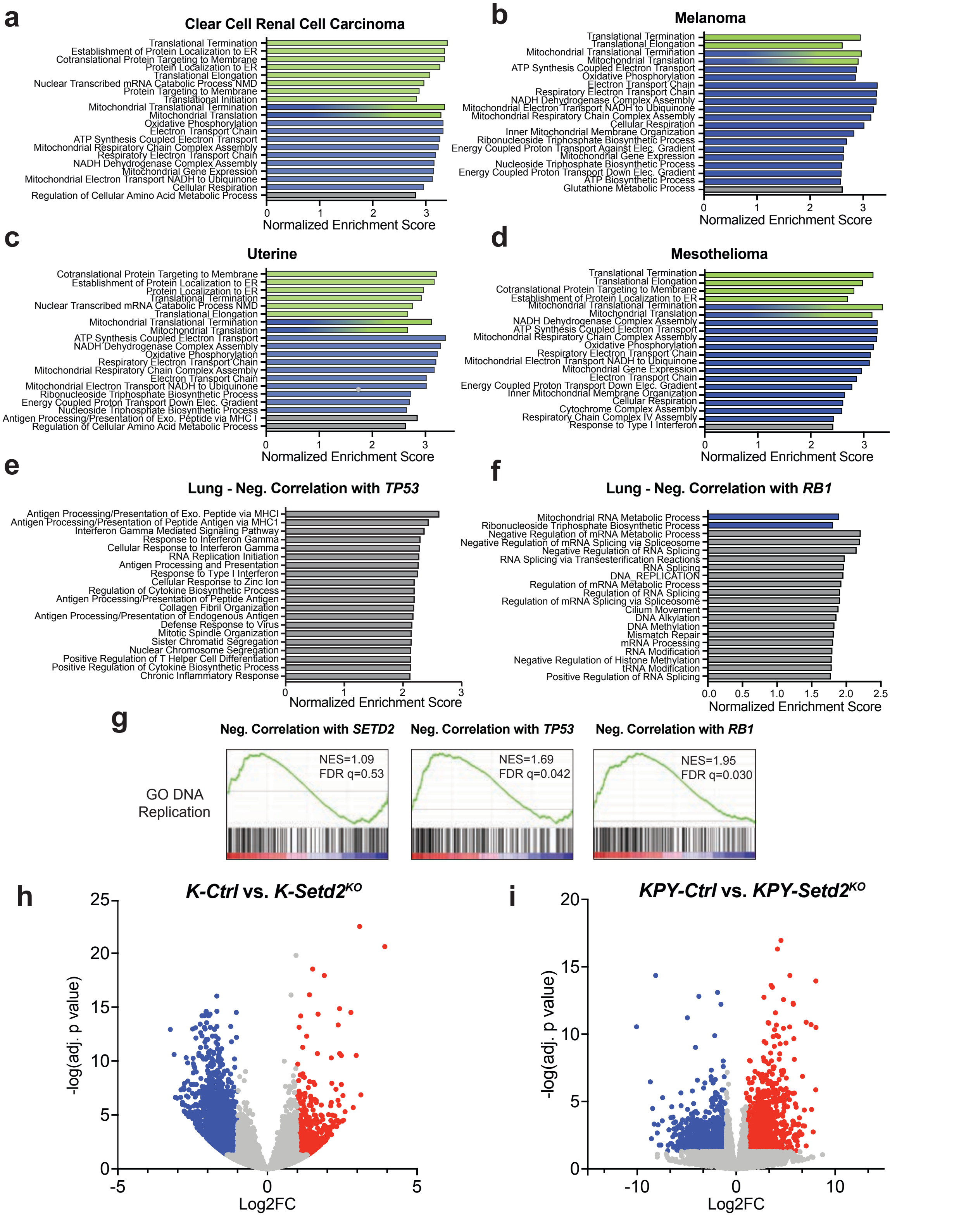

Supplementary Fig. 1: SETD2 deficiency promotes protein synthesis and OXPHOS gene expression programs in multiple human cancer types. Gene sets containing both mitochondrial and ribosomal genes are blue with green diagonal lines. The size of the bar indicates the normalized enrichment score (NES) of the gene set. **a)** The top 20 gene sets in human clear cell renal cell carcinoma correlated with low expression of *SETD2*. **b)** The top 20 gene sets in human melanoma correlated with low expression of *SETD2*. **c)** The top 20 gene sets in human uterine cancer correlated with low expression of *SETD2*. **d)** The top 20 gene sets in human mesothelioma correlated with low expression of *SETD2*. **e)** The top 20 gene sets in human lung adenocarcinoma correlated with low expression of *TP53*. **f)** The top 20 gene sets in human lung adenocarcinoma correlated with low expression of *RB1*. **g)** Gene set enrichment analysis plots of the GO DNA Replication gene negatively correlated with *SETD2*, *TP53* or *RB1* gene expression in human lung adenocarcinomas. **h,i)** Volcano plots of RNA-sequencing data in **h)** *K-Ctrl* vs *K-Setd2^{KO}* tumors and **i)** *KPY-Ctrl* vs *KPY-Setd2^{KO}* tumors. Colored dots represent genes that are differentially enriched (log2 fold-change greater-than 1 and false discovery rate (FDR)-adjusted P-value less-than 0.05)

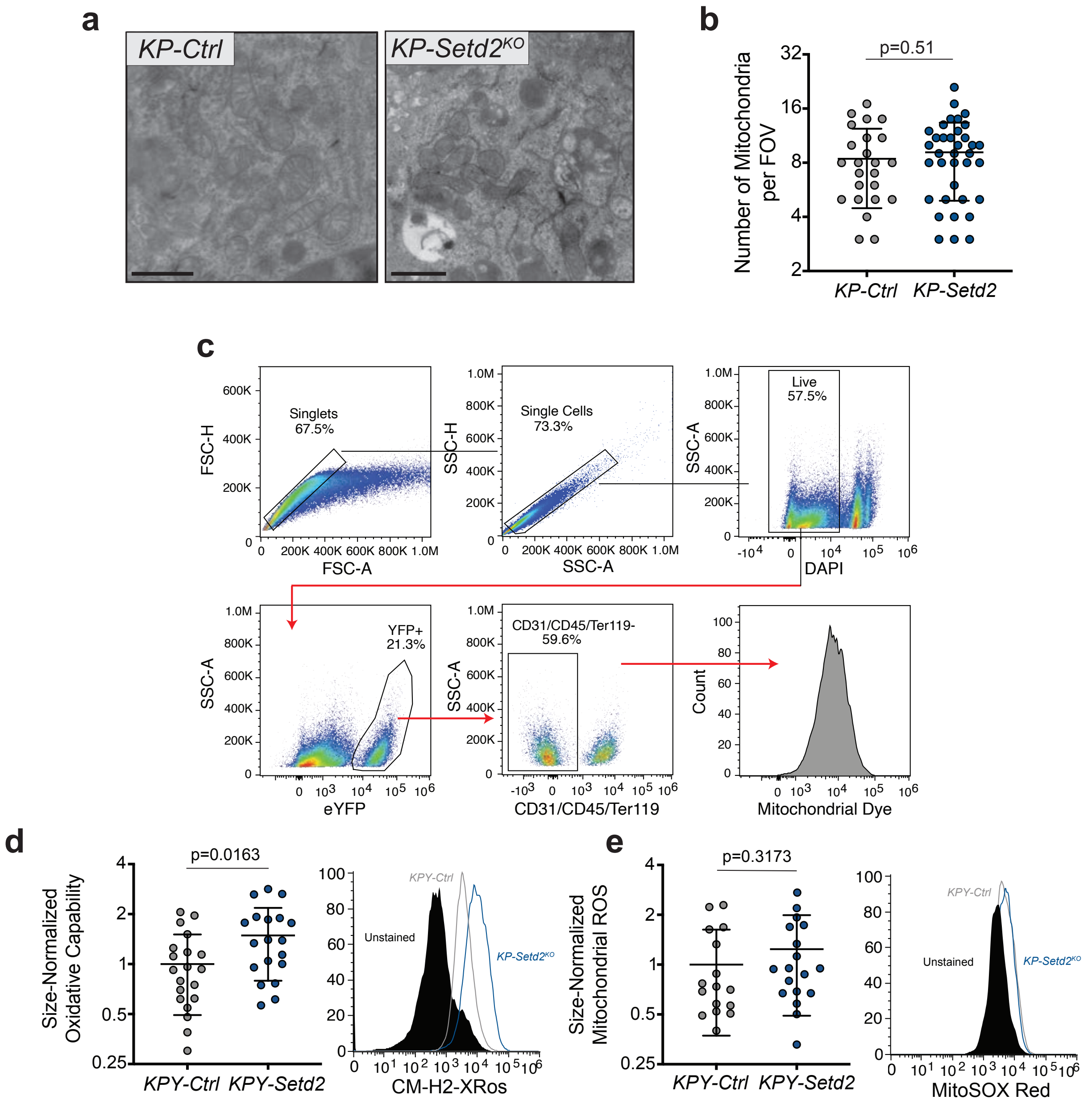

Supplementary Fig. 2: Characterization of mitochondrial properties in *KP(Y)*-Ctrl and *KP(Y)- Setd2^{KO}* tumors. a) Representative EM photomicrographs demonstrating that *KP-Setd2^{KO}* tumors have similar numbers of mitochondria, that are smaller and more electron dense, than *KP-Ctrl* tumors. Scale bars =2 µm. **b)** Quantification of the number of mitochondria per field of view from electron micrographs of 15,000X magnification. Data indicate the mean ± standard deviation. Data points represent individual tumors (*KP-Ctrl*: n=24 micrographs, n=3 mice, *KP- Setd2^{KO}*: n=36 micrographs, n=3 mice). Significance determined by unpaired Student's *t*-test. **c)** Gating strategy for dye-based, *ex vivo* mitochondrial analyses in isolated tumor cells from *KPY* mice. Doublets are excluded based on FSC and SSC properties. DAPI- positive dead cells and YFP-negative cells are excluded. CD31/ CD45/Ter-119- positive non-epithelial cell types are excluded. Remaining YFP-positive tumor cells are analyzed for mitochondrial dye fluorescence intensity. **d)** Quantification of the oxidative capability of mitochondria within *KPY-Ctrl* and *KPY-Setd2^{KO}* tumor cells by the median fluorescence intensity of MitoTracker Red CM-H_2- XRos staining by flow cytometry and normalized to the mitochondrial mass of each sample. Data represent the mean ± standard deviation. Data points represent individual tumors (*KPY-Ctrl*: n=20 tumors, n=2 mice, *KPY-Setd2^{KO}*: n=19 tumors, n=2 mice). Significance determined by unpaired Student's *t*-test. Histogram shows representative flow data from *KPY*-Ctrl and *KPY-Setd2^{KO}* tumors with unstained control. **e)** Quantification of mitochondrial reactive oxygen species (ROS)/superoxide levels within *KPY-Ctrl* and *KPY-Setd2^{KO}* tumor cells by the median fluorescence intensity of MitoSOX Red staining by flow cytometry and normalized to the mitochondrial mass of each sample. Data represent the mean ± standard deviation. Data points represent individual tumors (*KPY-Ctrl*: n=16 tumors, n=2 mice, *KPY-Setd2^{KO}*: n=19 tumors, n=2 mice). Significance as determined by unpaired Student's *t*-test. Histogram shows representative flow data from *KPY*-Ctrl and *KPY-Setd2^{KO}* tumors with unstained control.

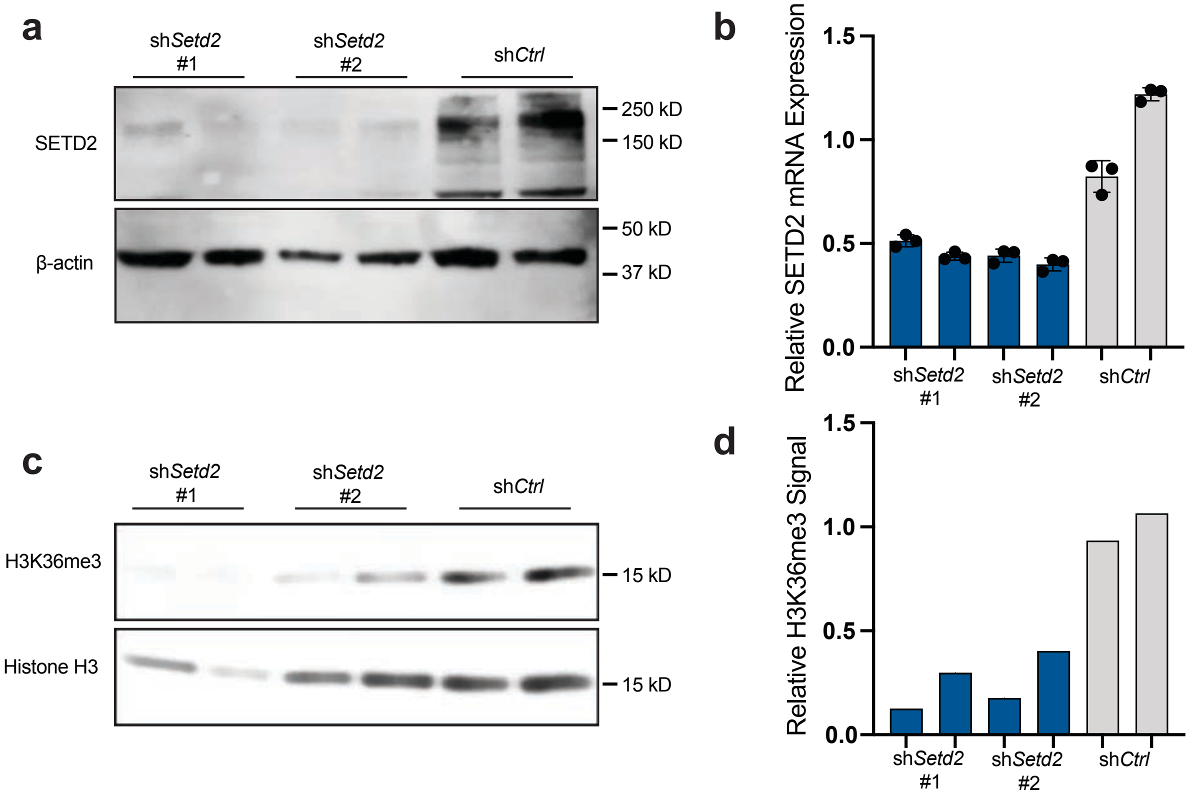

Supplementary Fig. 3 Generation of SETD2 shRNA human cell lines. a) Immunoblot analysis for SETD2 on whole-cell lysates derived from H2009 sh*Setd2* and sh*Ctrl* cell lines. β-actin is used as a loading control. **b)** Quantative real-time PCR for *Setd2* expression. Expression is normalized to β-actin and is relative to expression of *Setd2* in sh*Ctrl* cell lines. Data indicate the mean ± standard deviation. **c)** Immunoblot analysis on histones derived from H2009 sh*Setd2* and sh*Ctrl* cell lines for H3K36me3. Histone H3 is used as a loading control. **d)** Quantification of immunoblot from (a). H3K36me3 signal is normalized to total H3 for each lane.

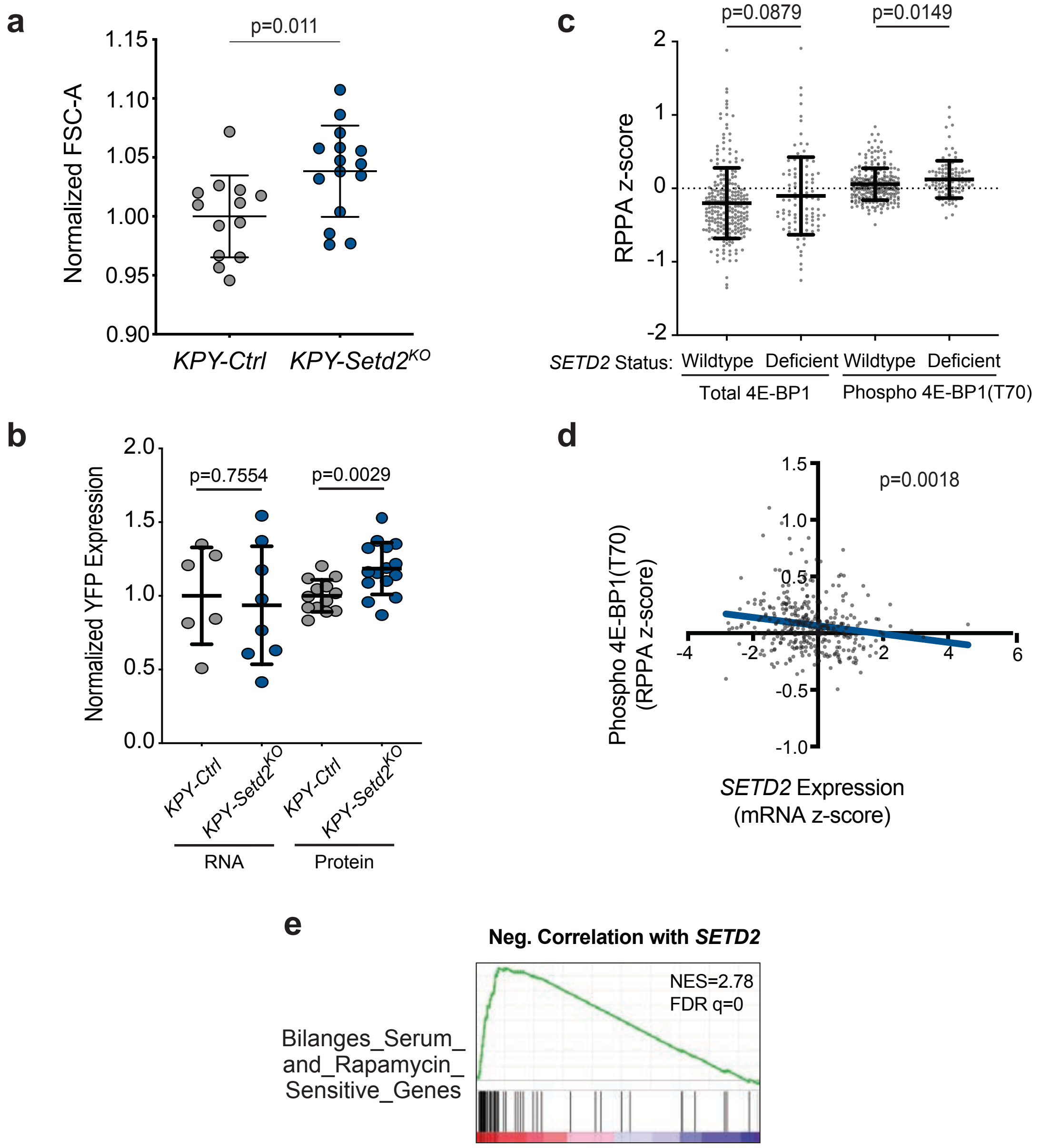

Supplementary Fig. 4: mTORC1 signaling and protein synthesis are heightened in SETD2-deficient tumors. a) Quantification of mean cell size in *KPY-Ctrl* and *KPY-Setd2*^{KO}tumors by forward scatter area (FSC-A) by flow cytometry. Data indicate the mean ± standard deviation. Data points represent individual tumors (*KPY-Ctrl*: n=13 tumors, n=7 mice, *KPY-Setd2*^{KO}: n=15 tumors, n=8 mice). Significance determined by unpaired Student's *t*- test. **b)** Normalized expression of YFP RNA and protein in *KPY-Ctrl* and *KPY-Setd2*^{KO} tumors. Data indicate the mean ± standard deviation. Data points represent individual tumors (RNA, *KPY-Ctrl*: n=6 tumors, n=4 mice, *KPY-Setd2*^{KO}: n=8 tumors, n=5 mice. Protein, *KPY-Ctrl*: n=13 tumors, n=7 mice, *KPY-Setd2*^{KO}: n=15 tumors, n=8 mice). Significance determined by unpaired Student's *t*-test. **c)** Quantification of total 4E-BP1 and phosphorylated 4E-BP1(T70) by RPPA in human lung adenocarcinomas with wildtype *SETD2* or *SETD2* deficiency. Data indicate the mean ± standard deviation. Data points represent individual patient tumors (wildtype *SETD2*: n=253 tumors, *SETD2* deficiency: n=103 tumors). Significance determined by unpaired Student's *t*-test. **d)** Analysis of the relationship between *SETD2* gene expression (mRNA z-score) and phosphorylated 4E-BP1(T70) as quantified by RPPA in human lung adenocarcinomas. Data points represent individual patient tumors (n=357 tumors). Significance determined by linear regression analysis and the line of best fit is shown. **e)** mTORC1 signaling-related gene set in human lung adenocarcinoma correlated with low expression of *SETD2*.

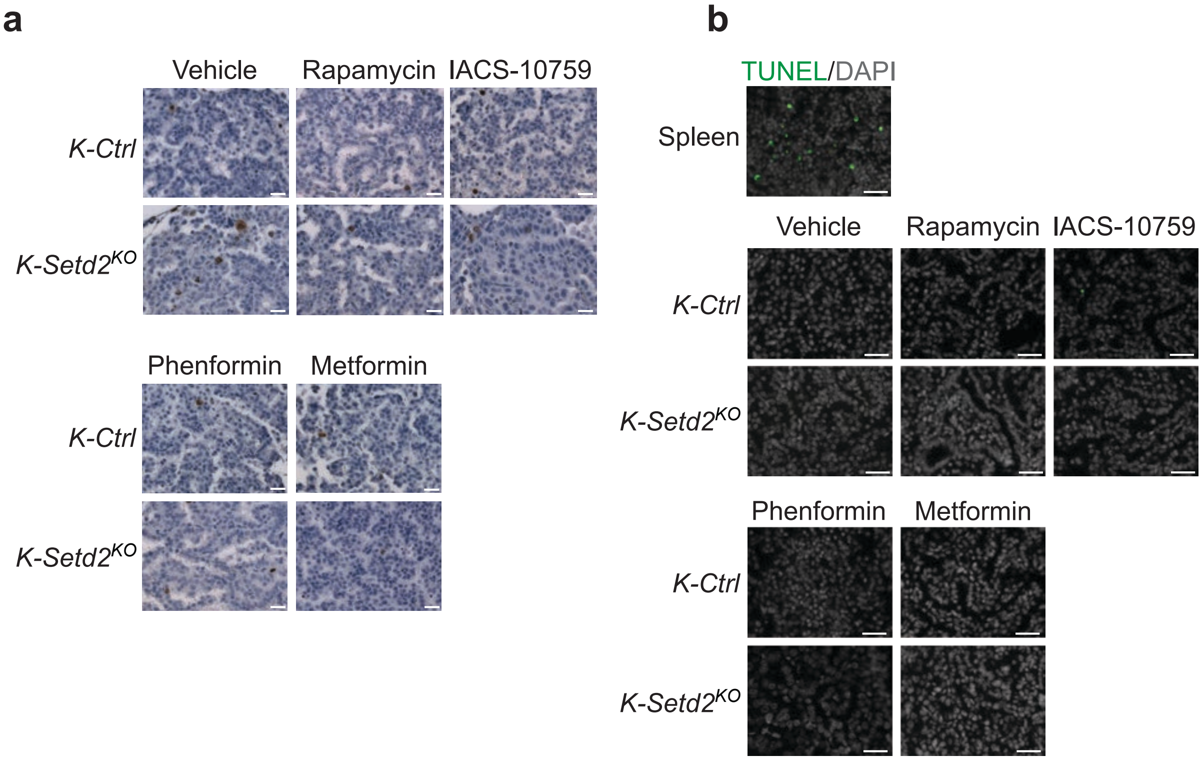

Supplementary Fig. 5: Inhibiting mTORC1 signaling or mitochondrial activity reduces cell proliferation in SETD2-deficient tumors. a) Representative images from p-H3 staining of *K-Ctrl* and *K-Setd2^{KO}* tumors from mice treated with vehicle, rapamycin, IACS-10759, phenformin or metformin. Scale bars= 25 µm. **b)** Representative images from TUNEL staining of *K-Ctrl* and *K-Setd2^{KO}* tumors from mice treated with vehicle, rapamycin, IACS-10759, phenformin or metformin. Positive control for TUNEL is a murine spleen. TUNEL is marked by green, DAPI-stained nuclei are in white. Scale bars= 300 µm.

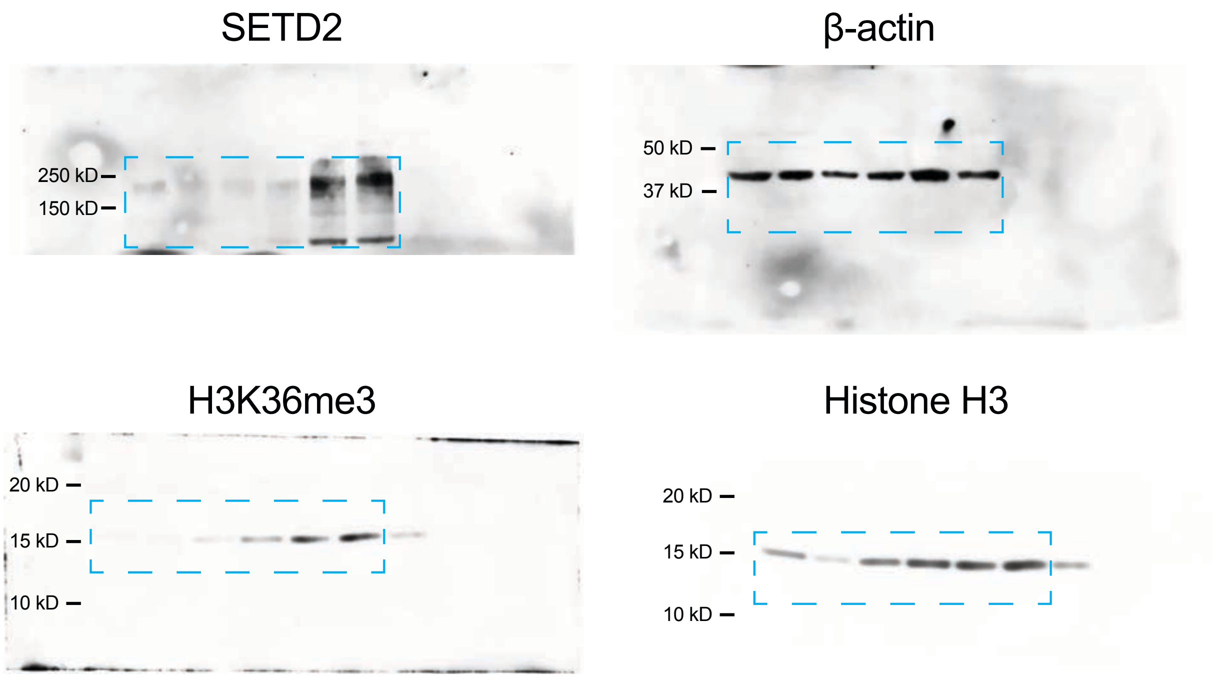

Supplementary Fig. 6: Compilation of uncropped immunoblots.
