# Supplementary material for: Setd2 inactivation sensitizes lung adenocarcinoma to inhibitors of oxidative respiration and mTORC1 signaling
Source: Commun Biol. 2023 Mar 10;6:255. doi: 10.1038/s42003-023-04618-3 (PMC10006211; doi:10.1038/s42003-023-04618-3)
Supplement: Supplementary file 3 — Description of Additional Supplementary Files [file 42003_2023_4618_MOESM3_ESM.pdf]

Description of Additional Supplementary Files

File name: Supplementary Data 1

Description: Differentially expressed genes from DESeq analysis performed on K-Ctrl vs KSetd2KO tumors and KPY-Ctrl vs KPY-Setd2KO tumors.

File name: Supplementary Data 2

Description: The source data behind the graphs in the paper.
