# Supplementary material for: Setd2 inactivation sensitizes lung adenocarcinoma to inhibitors of oxidative respiration and mTORC1 signaling
Source: Commun Biol. 2023 Mar 10;6:255. doi: 10.1038/s42003-023-04618-3 (PMC10006211; doi:10.1038/s42003-023-04618-3)
Supplement: Supplementary file 6 — Reporting Summary [file 42003_2023_4618_MOESM6_ESM.pdf]

nature portfolio

Corresponding author(s): David Feldser

Last updated by author(s): 02/08/2023

Reporting Summary

Nature Portfolio wishes to improve the reproducibility of the work that we publish. This form provides structure for consistency and transparency in reporting. For further information on Nature Portfolio policies, see our Editorial Policies and the Editorial Policy Checklist.

Statistics

For all statistical analyses, confirm that the following items are present in the figure legend, table legend, main text, or Methods section.

n/a	Confirmed	
☐	☒	The exact sample size (*n*) for each experimental group/condition, given as a discrete number and unit of measurement
☐	☒	A statement on whether measurements were taken from distinct samples or whether the same sample was measured repeatedly
☐	☒	The statistical test(s) used AND whether they are one- or two-sided<br>*Only common tests should be described solely by name; describe more complex techniques in the Methods section.*
☒	☐	A description of all covariates tested
☒	☐	A description of any assumptions or corrections, such as tests of normality and adjustment for multiple comparisons
☐	☒	A full description of the statistical parameters including central tendency (e.g. means) or other basic estimates (e.g. regression coefficient) AND variation (e.g. standard deviation) or associated estimates of uncertainty (e.g. confidence intervals)
☐	☒	For null hypothesis testing, the test statistic (e.g. *F*, *t*, *r*) with confidence intervals, effect sizes, degrees of freedom and *P* value noted<br>*Give P values as exact values whenever suitable.*
☒	☐	For Bayesian analysis, information on the choice of priors and Markov chain Monte Carlo settings
☒	☐	For hierarchical and complex designs, identification of the appropriate level for tests and full reporting of outcomes
☒	☐	Estimates of effect sizes (e.g. Cohen's *d*, Pearson's *r*), indicating how they were calculated

Our web collection on statistics for biologists contains articles on many of the points above.

Software and code

Policy information about availability of computer code

Data collection	No custom or commercial code was used to collect data.
Data analysis	Prism Software package version 9 was used for all statistical analyses.

For manuscripts utilizing custom algorithms or software that are central to the research but not yet described in published literature, software must be made available to editors and reviewers. We strongly encourage code deposition in a community repository (e.g. GitHub). See the Nature Portfolio guidelines for submitting code & software for further information.

Data

Policy information about availability of data

All manuscripts must include a data availability statement. This statement should provide the following information, where applicable:

- Accession codes, unique identifiers, or web links for publicly available datasets
- A description of any restrictions on data availability
- For clinical datasets or third party data, please ensure that the statement adheres to our policy

The data supporting the findings of the study are available within the paper and its supplementary information files and have been deposited publicly in the Gene Expression Omnibus under accession number GSE224270.

Human research participants

Policy information about studies involving human research participants and Sex and Gender in Research.

Reporting on sex and gender	Our study did not use human research participants.
Population characteristics	Our study did not use human research participants.
Recruitment	Our study did not use human research participants.
Ethics oversight	Our study did not use human research participants.

Note that full information on the approval of the study protocol must also be provided in the manuscript.

Field-specific reporting

Please select the one below that is the best fit for your research. If you are not sure, read the appropriate sections before making your selection.

☒ Life sciences ☐ Behavioural & social sciences ☐ Ecological, evolutionary & environmental sciences

For a reference copy of the document with all sections, see nature.com/documents/nr-reporting-summary-flat.pdf

Life sciences study design

All studies must disclose on these points even when the disclosure is negative.

Sample size	Commonly, we tried to randomize ~10 mice into each group. Given the heterogeneity in tumors, combined with the number of mice, we found adequate power for analysis.
Data exclusions	Outliers were excluded using the ROUT method with a Q of 0.1%.
Replication	Animals were enrolled into in vivo experiments as they became available until sufficient numbers were obtained to determine experimental outcomes.
Randomization	Allocation of mice into experimental groups was random.
Blinding	Investigators were not blinded when conducting data collection.

Reporting for specific materials, systems and methods

We require information from authors about some types of materials, experimental systems and methods used in many studies. Here, indicate whether each material, system or method listed is relevant to your study. If you are not sure if a list item applies to your research, read the appropriate section before selecting a response.

Materials & experimental systems

n/a	Involved in the study	
☐	☒	Antibodies
☐	☒	Eukaryotic cell lines
☒	☐	Palaeontology and archaeology
☐	☒	Animals and other organisms
☒	☐	Clinical data
☒	☐	Dual use research of concern

Methods

n/a	Involved in the study	
☒	☐	ChIP-seq
☐	☒	Flow cytometry
☒	☐	MRI-based neuroimaging

Antibodies

Antibodies used	Primary antibodies for immunostaining include the following: H3K36me3 (Abcam, ab9050, 1:1000), H3 (1:10000, Abcam ab1791), SETD2 (Cell Signaling Technology, E4W8Q, 1:1000), β-actin (1:10000,Sigma Aldrich, A2066), (p-4E-BP1(T37/46) (Cell Signaling Technology, cs2855, 1:100), mTOR (Cell Signaling Technology, cs2983, 1:100), LAMP2 (Abcam, ab13524, 1:100), and p-H3 (Cell Signaling Technology, cs9701, 1:500). P-4E-BP1(T37/46) was assessed by immunofluorescence using a biotinylated secondary antibody (Vector Laboratories, PK-4001) according to product instructions, and Streptavidin-conjugated Alexa594 (Thermo Fisher S11227, 1:200). Colocalization of mTOR and LAMP2 was determined using an anti-Rat Alexa647 antibody (Thermo Fisher A21247,

	1:200) to detect LAMP2, and a biotinylated anti-Rabbit secondary antibody (Vector Laboratories, PK-4001) followed by Streptavidin-conjugated Alexa488 (Thermo Fisher S32354, 1:200) to detect mTOR. To quantify mitochondrial ROS cells were incubated with 5 µM MitoSOX Red (Thermo Fisher, M36008). For flow cytometry analysis, biotinylated antibodies against CD31 (BD Biosciences, 558737, 1:100), CD45 (BD Biosciences, 553078, 1:200), and Ter-119 (BD Biosciences, 553672, 1:100) were used to exclude non-epitheleal cell types. Streptavidin-conjugated APC- eFluor 780 (Thermo Fisher, 47-4317-82) was used to detect these antibodies.
Validation	All antibodies were used in accordance to the manufacturer guidelines and have been well documented in the literature.

Eukaryotic cell lines

Policy information about cell lines and Sex and Gender in Research

Cell line source(s)	Human lung adenocarcinoma cell line H2009 was obtained from the NCI cell line repository.
Authentication	Cell line was obtained from the NCI cell line repository and used in accordance to manufacturer guidelines.
Mycoplasma contamination	All human cell lines were tested by the NCI cell line repository and subsequently tested by our laboratory.
Commonly misidentified lines (See ICLAC register)	We have not utilized any misidentified cell lines in our study.

Animals and other research organisms

Policy information about studies involving animals; ARRIVE guidelines recommended for reporting animal research, and Sex and Gender in Research

Laboratory animals	Species = Mus musculus<br>Strain and Sex = GEM models are maintained on a mixed 129sv/Jae, C57Bl6J background (Male and Female), tumors initiated by 20 weeks of age.
Wild animals	Study did not involve wild animals.
Reporting on sex	In vivo experiments were sex balanced, to the best of our ability.
Field-collected samples	Our study did not involve field-collected samples.
Ethics oversight	Our animal protocol was approved by the University Laboratory Animal Resources (ULAR) at the University of Pennsylvania and the IACUC.

Note that full information on the approval of the study protocol must also be provided in the manuscript.

Flow Cytometry

Plots

Confirm that:

☒ The axis labels state the marker and fluorochrome used (e.g. CD4-FITC).

☒ The axis scales are clearly visible. Include numbers along axes only for bottom left plot of group (a 'group' is an analysis of identical markers).

☒ All plots are contour plots with outliers or pseudocolor plots.

☒ A numerical value for number of cells or percentage (with statistics) is provided.

Methodology

Sample preparation	Tumors were microdissected directly from the lungs of KPY mice and individually placed in 500 µl of tumor digestion buffer consisting of PBS containing 10 mM HEPES pH 7.4, 150 mM NaCl, 5 mM KCl, 1 mM MgCl2, and 1.8 mM CaCl2, along with freshly added Collagenase 4 (Worthington 100 mg/ml solution, 20 µl per ml of digestion buffer) and DNase I (Roche 10 mg/ml solution, 4 µl per ml of digestion buffer). Tumors were manually disassociated using scissors, and then placed in a 4 °C shaker for 1 hour at 250 rpm. Digested tumors were then filtered into strainer-cap flow tubes (Corning, 352235) containing 1 ml of horse serum (Thermo Fisher, 16050122) to quench the digestion reaction. Cells were spun down at 200 g for 5 minutes with the cap in place to obtain all cells.
Instrument	Thermo Fisher Scientific Attune NxT Flow Cytometer
Software	Flow cytometry data was analyzed using FlowJo software.
Cell population abundance	Reported as the percentage of total events after gating out debris.

Gating strategy

Single, viable cells were determined by gating out debris, dead cells, and clumps using FSC/SSC.

☒ Tick this box to confirm that a figure exemplifying the gating strategy is provided in the Supplementary Information.
